# Associations between interrelated dimensions of socio-economic status, higher risk drinking and mental health in South East London: A cross-sectional study

**DOI:** 10.1371/journal.pone.0229093

**Published:** 2020-02-14

**Authors:** Sadie Boniface, Dan Lewer, Stephani L. Hatch, Laura Goodwin

**Affiliations:** 1 Institute of Alcohol Studies, Alliance House, London, United Kingdom; 2 Addictions Department, Institute of Psychiatry, Psychology & Neuroscience, King’s College London, London, United Kingdom; 3 Institute of Epidemiology and Health Care, University College London, London, United Kingdom; 4 Department of Psychological Medicine, Institute of Psychiatry, Psychology & Neuroscience, King’s College London, London, United Kingdom; 5 Department of Psychological Sciences, University of Liverpool, Liverpool, United Kingdom; University of Sao Paulo Medical School, BRAZIL

## Abstract

**Aim:**

To examine patterns of hazardous, harmful and dependent drinking across different socio-economic groups, and how this relationship may be explained by common mental disorder.

**Methods and findings:**

Between 2011–2013, 1,052 participants (age range 17–91, 53% female) were interviewed for Phase 2 of the South East London Community Health study. Latent class analysis was used to define six groups based on multiple indicators of socio-economic status in three domains. Alcohol use (low risk, hazardous, harmful/dependent) was measured using the Alcohol Use Disorders Identification Test and the presence of common mental disorder was measured using the revised Clinical Interview Schedule. Multinomial regression was used to explore associations with hazardous, harmful and dependent alcohol use, including after adjustment for common mental disorder.

Harmful and dependent drinking was more common among people in Class 2 ‘economically inactive renters’ (relative risk ratio (RRR) 3.05, 95% confidence interval (CI) 1.07–8.71), Class 3 ‘economically inactive homeowners’ (RRR 4.11, 95% CI 1.19–14.20) and Class 6 ‘professional renters’ (RRR 3.51, 95% CI 1.14–10.78) than in Class 1 ‘professional homeowners’. Prevalent common mental disorder explained some of the increased risk of harmful or dependent drinking in Class 2, but not Class 3 or 6.

**Conclusions:**

Across distinct socio-economic groups in a large inner-city sample, we found important differences in harmful and dependent drinking, only some of which were explained by common mental disorder. The increased risk of harmful or dependent drinking across classes which are very distinct from each other suggests differing underlying drivers of drinking across these groups. A nuanced understanding of alcohol use and problems is necessary to understand the inequalities in alcohol harms.

## Introduction

Globally there are close to 2 billion people who drink alcohol [1]. Alcohol is the seventh leading risk factor in terms of deaths and disability-adjusted life years lost [2], with alcohol use disorders leading to 11 million healthy years of life lost annually worldwide [3]. In England, alcohol contributes to 8,000 deaths and a million hospital admissions annually, and although consumption patterns appear stable, hospital admissions are rising [4].

Alcohol harm disproportionately affects the most disadvantaged socio-economic groups despite surveys consistently identifying that people belonging to higher socio-economic groups report drinking the same or more on average. This has been observed repeatedly in the UK [5–8] as well as a number of other European countries [9,10] and Australia [11,12]. Many explanations exist for this ‘alcohol harm paradox’, one of which is very heavy or problematic drinking being clustered in lower socio-economic groups, which is supported by recent research in the UK [5,6].

As with alcohol harms there are also inequalities in mental health, with evidence for strong social patterning of common mental disorders (CMDs) such as anxiety and depression [13]. CMDs are highly comorbid with alcohol use disorders and many individuals with a mental health problem report drinking heavily to cope with their symptoms [14]. There is also a vast literature on the reduced life expectancy of individuals with mental disorders [15], so it is important to consider the role of mental health in inequalities in alcohol use and harm.

Many surveys measure socio-economic status (SES) using income, occupational grade, employment status, education, housing and area deprivation. Studies of social patterning in drinking behaviour have used these measures individually, which can identify social gradients in behaviours but may miss groups that are better defined by combinations of these variables. Drinking behaviours vary substantially within groups defined by traditional markers of socioeconomic status, and there may be social groups that are poorly defined by these markers. A small number of recent studies have developed composite scores [5,16], which have the advantages of weighting different dimensions of SES according to their importance and accounting for the overlapping nature of aspects of SES. However, interpretation of results using composite SES measures is less straightforward, and composite scores can also mask the patterns in SES and drinking relationships that are useful to allow substantive conclusions to be drawn about these relationships.

One statistical approach to examining how multiple aspects of social disadvantage (or advantage) overlap is latent class analysis (LCA). This is best described by the concept of intersectionality, which arose in black feminist theory [17] and is used to describe the experience of multiple aspects of social disadvantage in relation to characteristics including (but not limited to) gender, race, socio-economic status, sexual orientation, age or disability. LCA is a type of mixture modelling and a commonly used intersectional quantitative analysis method. In LCA, classes or groups of individuals with similar characteristics are defined, and then factors such as health behaviours or health outcomes can be looked at in relation to class membership, making LCA a person-centred approach to understanding population heterogeneity. LCA has been used to study experiences of multiple positions of social privilege or disadvantage that can co-occur [18–21]. Previous work by our group has involved a LCA of socio-economic groups in the current sample and identified nuance in the association between these classes with CMD, with the prevalence of CMD highest in a class characterised by multiple levels of disadvantage [21].

The current study will show whether drinking behaviours can be better explained using a categorical approach to socioeconomic status based on LCA, rather than traditional approaches based on ordinal measures of social status. Through taking into account intersectionality in SES, it may be possible to better understand patterns in drinking, and consequently to improve targeting of public health interventions. Moreover, previous work has indicated the importance of understanding patterns at local as well as national scales [21,22], however most alcohol inequalities research has been on a national level.

The aim of this study is to examine patterns in hazardous, harmful and dependent drinking across different socio-economic groups (as defined in [21]) and how these relationships may be explained by CMD in the inner-city South East London Community Health (SELCoH) study (Phase 2).

## Methods

### Data source and ethical approval

SELCoH is a longitudinal community survey of people living in randomly sampled households in two boroughs in South East London (Lambeth and Southwark), which assesses demographic and socioeconomic characteristics; physical and mental health symptoms; health service use; and a range of social stressors and psychosocial resources [23,24]. SELCoH I included 1,698 adults from 1075 randomly selected households interviewed from 2008 to 2010 (household participation rate 51.9%, within-household participation rate 71.9%). The data are available to researchers through the NIHR Maudsley BRC by contacting selcoh@kcl.ac.uk. SELCoH II targeted 1,596 participants who agreed to be re-contacted and 1,052 were interviewed between 2011 and 2013 (response rate 73%) and were analysed in the current study. SELCoH II was chosen for this analysis as it is the most recent wave available.

Ethical approval for SELCoH II was received from the King’s College London Psychiatry, Nursing and Midwifery Research Ethics Committee (PNM/10/11-106). Written consent was obtained from participants. This study did not have a published analysis protocol. STROBE reporting guidelines were followed [25].

### Measures

The socio-economic indicators used to identify the latent classes were the same as those used in a recent paper by Goodwin and colleagues [21]. The indicators were in three groups, income and occupation, housing status, and education level. The income and occupation indicators included: (1) gross annual household income, collapsed into three categories (£0–£12,097, £12,098–£31,494, £31,495+), (2) employment status, categorised into full or part-time employment; student; unemployed; and other (including sick, disabled, retired or carer), (3) occupational social grade (SOC) according to the Registrar General’s classification, collapsed into four categories: professional & managerial (classes I and II); skilled (class III non- manual and manual); semi-skilled and unskilled (classes IV and V); and no SOC assigned, (4) current benefit receipt (excluding state pension and child benefit), and (5) debt in the past year (excluding mortgage). The housing status indicators included: (1) number of times the participant had moved in the past 2 years (0–1 times or 2+ times), and (2) housing tenure in four categories (own outright/mortgage, private rented, social housing, rent free). The educational level indicator was highest qualification obtained by the participant, in three categories (no qualifications/GCSE, A-level, degree or above).

Alcohol use was measured using the Alcohol Use Disorders Identification Test (AUDIT), a widely-used 10-item screening tool developed by WHO [26]. As is common practice, the following AUDIT score cut points were used to categorise drinking risk levels: 0–7 = low risk, 8–15 = hazardous, 16–19 = harmful, 20+ probable dependence. Due to small numbers of harmful and dependent drinkers in the dataset, the two highest risk categories were collapsed, and a categorical variable with three values (low risk, hazardous, harmful/dependent) was used in the multinomial regression. The sample proportions by each individual socio-economic indicator and by low risk, hazardous and harmful/dependent drinking were calculated (S1 Table).

Common mental disorder (CMD) was measured using the revised Clinical Interview Schedule (CIS-R) which covers 14 symptom domains: fatigue, sleep problems, irritability, worry, depression, depressive ideas, anxiety, obsessions, subjective memory and concentration, somatic symptoms, compulsions, phobias, physical health worries and panic [27]. A score ≥ 12 indicated presence of a CMD as used widely including in previous SELCoH studies [21].

### Class enumeration

Latent class analysis was used to identify discrete classes based on income, occupation, housing and education. A previously published six class solution identified by Goodwin and colleagues was reproduced in Mplus version 7.3 and used for subsequent analyses (for full details of class enumeration and model selection, see [21]). The estimated proportion in each class and the modal class assignment proportion along with the average posterior class probability and odds of correct classification are shown in Table 1.

**Table 1:** Summary of six class solution from latent class analysis with size and model fit statistics.

	Estimated proportion	90% CI	Modal Class Assignment Proportion (mcaP)	Average posterior class probability (AvePP)	Odds of correct classification (OCC)
Class 1	0.324	0.284–0.365	0.324	0.938	31.498
Class 2	0.194	0.168–0.219	0.199	0.936	60.956
Class 3	0.083	0.051–0.115	0.080	0.914	117.357
Class 4	0.228	0.192–0.263	0.227	0.913	35.620
Class 5	0.123	0.086–0.161	0.121	0.931	95.805
Class 6	0.048	0.022–0.074	0.048	0.898	174.764

CI = confidence interval. Entropy = 0.898

### Statistical analysis

The six-class solution was reproduced from our group’s earlier study [21] and the modal class assignment proportion was inspected along with estimated proportion assigned to that class and the 90% confidence intervals. The average posterior class probability and odds of correct classification were calculated and inspected to confirm the fit of the six-class solution.

The gold-standard method for conducting analysis of categorical distal outcomes in a latent class analysis is using the DCAT auxiliary command (in Mplus), however this is not compatible with survey weights or including covariates in the model. After inspection of the probability of assignment to each of the six classes and the average posterior class probability the modal class assignment was exported from Mplus to Stata and a sensitivity analysis was conducted to explore the impact of including the probability of class assignment as a probability weight [28]. These regression models showed that weighting the data by the probability of class assignment had a negligible impact on effect estimates and no impact on statistical significance (S2 Table), therefore it was preferential to choose the procedure where sampling and response weights could be included. This sensitivity analysis confirmed it was appropriate to proceed using modal class assignment as the exposure variable in Stata, and incorporating covariates as well as sampling and response weights into the analysis.

Multinomial logistic regression was used to assess the association between class membership and hazardous and harmful risk/dependent (combined due to small numbers) drinking before and after adjustment for CMD, with reference categories of Class 1 (professional homeowners–the largest class) and low risk drinking. There was little missing data and complete case analysis was used. The data were weighted to account for clustering by household and for within-household non-response and sample attrition between SELCoH I and SELCoH II. Analyses were adjusted for age, gender, ethnicity, marital status and number of children as all of these have known associations with alcohol use. The predicted probability of hazardous, harmful and dependent drinking across the different classes was calculated using the ‘margins’ postestimation command. Regression analyses were conducted in Stata version 15.

## Results

There were 1,052 people interviewed between 2011–13. The overall sample characteristics are shown in Table 2.

**Table 2:** Overall sample characteristics.

		N (%)
Gender	Male	499 (47)
	Female	553 (53)
Age	16–24	184 (18)
	25–34	277 (26)
	35–44	201 (19)
	45–54	174 (17)
	55–64	117 (11)
	65+	99 (9)
Ethnic group	White British	523 (50)
	Black Caribbean	88 (8)
	Black African	141 (13)
	White Other	143 (14)
	Non-White Other	100 (10)
	Mixed	57 (5)
Marital Status	Single	445 (42)
	Married/Cohabiting	537 (51)
	Divorced/Separated/Widowed	69 (7)
Number of children	No children	484 (46)
	1–2 children	375 (36)
	3+ children	193 (18)

Data from 1,052 adults, weighted to account for complex survey design and non-response

The sample proportions by each socio-economic indicator and by low risk, hazardous and harmful/dependent drinking are provided in S1 Table. Briefly, hazardous drinking was more common among individuals who were employed, in the highest occupational grade and with the highest educational qualifications. Harmful and dependent drinking was more common among individuals not in work, in debt, and with less secure housing.

**Fig 1:**
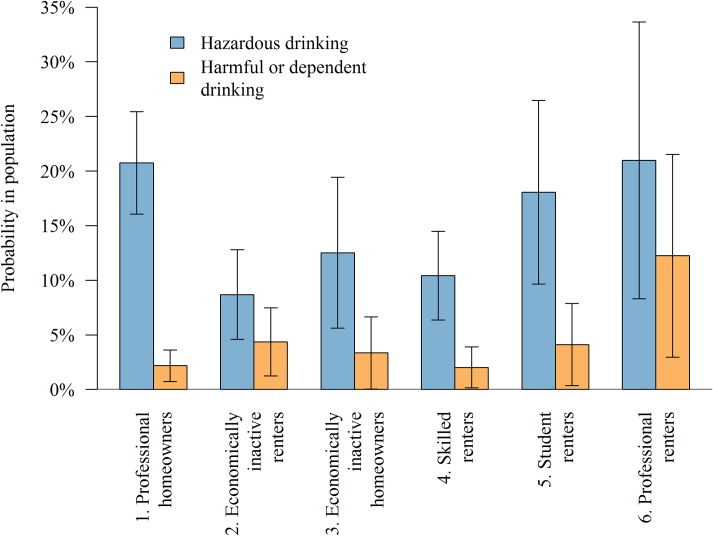
Probability of hazardous and harmful drinking by class, predicted from multinomial regression analysis including age, sex, ethnicity, marital status and number of children, with 95% CIs.

A description of each of the six classes identified using multiple SES indicators is shown in Table 3. The predicted probabilities of hazardous and harmful or dependent drinking across the different classes is shown in Fig 1 (predicted from the adjusted multinomial regression).

**Table 3:** Description of the six classes identified in the latent class analysis.

		CMD prevalence taken from Goodwin 2017
Class 1	***Professional homeowners*, *32% sample***	13.8% (‘Class 1’)
	All in work and 85% in professional/managerial roles. Little benefit receipt or debt	
	Majority homeowners (67%) and most of rest private renters	
	High education level (91% degree or higher)	
Class 2	***Economically inactive renters*, *19% sample***	41.5% (‘Class 5’)
	High levels of benefit receipt (76%) and debt (32%). Majority sick/disabled/retired/carer (64%), remainder unemployed.	
	All renting, mostly social housing (83%)	
	Low education levels—61% no quals/GCSEs	
Class 3	***Economically inactive homeowners*, *8% sample***	16.9% (‘Class 6’)
	High household income (61% in top group), but economically inactive with 84% sick/disabled/retired/carer and 13% unemployed, little benefit receipt and no debt	
	Mostly homeowners (88%)	
	High education level (67% degree or higher)	
Class 4	***Skilled renters*, *23% sample***	20.0% (‘Class 3’)
	Medium-high incomes (38% in top group, 46% in middle group) and all in work. Mixed occupational grades.	
	Moderate levels of benefit receipt and dept (~25%)	
	All levels of education represented	
Class 5	***Student renters*, *12% sample***	25.0% (‘Class 4’)
	High household income (67% in top group), majority students (75%) and rest unemployed. Some benefit receipt (15%) and debt (18%)	
	Mixed tenure	
	High education level (67% degree or higher)	
Class 6	***Professional renters*, *5% sample***	10.3% (‘Class 2’)
	All in work and 64% in professional/managerial occupations. High household incomes (81% in top category). Little benefit receipt or debt.	
	Mostly private renters (84%)	* *
	High education level (81% degree or higher)	*** ***

Regarding hazardous drinking, in the unadjusted model (Table 4) Class 2 ‘economically inactive renters’ and Class 4 ‘skilled renters’ both had around half the risk of drinking at hazardous levels compared with Class 1 ‘professional homeowners’ (RRR 0.46, 95% CI 0.27–0.80, P = 0.005 and RRR 0.52, 95% CI 0.31–0.87, P = 0.013 respectively). However these associations were not significant in the adjusted models.

**Table 4:** Multinomial logistic regression of the association between socio-economic status latent class membership and AUDIT category.

				Unadjusted					Adjusted for confounders*					Additionally adjusted for CMD*				
		Prob.	n	RRR	SE	Lower 95% CI	Upper 95% CI	P- value	RRR	SE	Lower 95% CI	Upper 95% CI	P-value	RRR	SE	Lower 95% CI	Upper 95% CI	P-value
**CLASS 1**	*Professional homeowners*, *32% sample*																	
	Low risk drinkers	0.76	265	1.00	0.00	1.00	1.00		1.00	0.00	1.00	1.00		1.00	0.00	1.00	1.00	
	Hazardous	0.21	74	1.00	0.00	1.00	1.00		1.00	0.00	1.00	1.00		1.00	0.00	1.00	1.00	
	Harmful/dependent	0.03	10	1.00	0.00	1.00	1.00		1.00	0.00	1.00	1.00		1.00	0.00	1.00	1.00	
**CLASS 2**	*Economically inactive renters*, *19% sample*																	
	Low risk drinkers	0.84	183	1.00	0.00	1.00	1.00		1.00	0.00	1.00	1.00		1.00	0.00	1.00	1.00	
	Hazardous	0.11	23	**0.46**	**0.13**	**0.27**	**0.80**	**0.005**	0.84	0.29	0.43	1.65	0.612	0.71	0.25	0.35	1.45	0.351
	Harmful/dependent	0.06	13	1.95	0.87	0.81	4.70	0.135	**3.05**	**1.63**	**1.07**	**8.71**	**0.037**	1.71	0.96	0.57	5.14	0.335
**CLASS 3**	*Economically inactive homeowners*, *8% sample*																	
	Low risk drinkers	0.82	80	1.00	0.00	1.00	1.00		1.00	0.00	1.00	1.00		1.00	0.00	1.00	1.00	
	Hazardous	0.13	13	0.61	0.20	0.32	1.17	0.137	1.57	0.56	0.78	3.16	0.203	1.56	0.55	0.78	3.13	0.211
	Harmful/dependent	0.04	4	1.34	0.74	0.45	3.94	0.599	**4.11**	**2.60**	**1.19**	**14.20**	**0.026**	**4.18**	**2.66**	**1.20**	**14.59**	**0.025**
**CLASS 4**	*Skilled renters*, *23% sample*																	
	Low risk drinkers	0.85	208	1.00	0.00	1.00	1.00		1.00	0.00	1.00	1.00		1.00	0.00	1.00	1.00	
	Hazardous	0.12	29	**0.52**	**0.14**	**0.31**	**0.87**	**0.013**	0.68	0.19	0.39	1.16	0.157	0.66	0.18	0.38	1.14	0.133
	Harmful/dependent	0.03	8	1.05	0.52	0.40	2.78	0.917	1.09	0.62	0.36	3.28	0.881	0.91	0.54	0.29	2.89	0.879
**CLASS 5**	*Student renters*, *12% sample*																	
	Low risk drinkers	0.74	74	1.00	0.00	1.00	1.00		1.00	0.00	1.00	1.00		1.00	0.00	1.00	1.00	
	Hazardous	0.21	21	0.89	0.25	0.51	1.56	0.685	0.63	0.23	0.31	1.28	0.203	0.60	0.22	0.29	1.21	0.152
	Harmful/dependent	0.05	5	1.73	0.98	0.57	5.28	0.336	0.77	0.48	0.23	2.65	0.678	0.61	0.40	0.17	2.18	0.450
**CLASS 6**	*Professional renters*, *5% sample*																	
	Low risk drinkers	0.66	29	1.00	0.00	1.00	1.00		1.00	0.00	1.00	1.00		1.00	0.00	1.00	1.00	
	Hazardous	0.20	9	1.02	0.46	0.43	2.45	0.961	0.88	0.40	0.37	2.14	0.783	0.90	0.41	0.37	2.21	0.820
	Harmful/dependent	0.14	6	**5.35**	**2.99**	**1.78**	**16.04**	**0.003**	**3.51**	**2.01**	**1.14**	**10.78**	**0.028**	**4.01**	**2.31**	**1.29**	**12.45**	**0.016**

*Adjusted for age, gender, ethnicity, marital status and number of children. RRR = relative risk ratio, SE = standard error, CI = confidence interval. Figures in bold statistically significant at the 5% level

In terms of harmful or dependent drinking, in the unadjusted model, Class 6 ‘professional renters’ had over five times the risk of harmful or dependent drinking compared with Class 1 ‘professional homeowners’ (RRR 5.35, 95% CI 1.78–16.04, P = 0.003). In the model adjusted for covariates, Class 2 ‘economically inactive renters’ (RRR 3.05, 95% CI 1.07–8.71, P = 0.037), Class 3 ‘economically inactive homeowners’ (RRR 4.11, 95% CI 1.19–14.20, P = 0.026) and Class 6 ‘professional renters’ (RRR 3.51, 95% CI 1.14–10.78, P = 0.028) all had significantly higher likelihood of drinking at this level compared with Class 1 ‘professional homeowners’.

After entering CMD into the model as a covariate, the effect estimates generally altered slightly, with the exception that Class 2 ‘economically inactive renters’ no longer had a significantly increased risk of harmful or dependent drinking, suggesting the higher CMD prevalence in this class explained the increased risk of harmful or dependent drinking.

## Discussion

This study took a person-centred approach to understand how multiple dimensions of social advantage and disadvantage are associated with higher risk drinking and CMD. We used a previously-identified 6-class solution to describe the patterns of socio-economic status and how they overlap in this diverse inner-city sample. These classes were substantively different and the multinomial regression identified some important differences in drinking risk levels across these classes. In the adjusted models, none of the classes had a significantly different odds of hazardous drinking compared with Class 1 ‘professional homeowners’. For harmful or dependent drinking, Class 2 ‘economically inactive renters’, Class 3 ‘economically inactive homeowners’ and Class 6 ‘professional renters’ all had between three and four times higher likelihood of drinking at this level compared with Class 1 ‘professional homeowners’. This increased odds was partly explained by the increased prevalence of CMD in Class 2 ‘economically inactive renters’ but not in Class 3 ‘economically inactive homeowners’ and Class 6 ‘professional renters’.

We chose Class 1 ‘professional homeowners’ as the reference category in this analysis because it was the largest class and it was broadly-speaking the most socio-economically advantaged overall. In this sample, none of the classes experienced a significantly different risk of drinking at hazardous levels than Class 1 ‘professional homeowners’. This runs counter to some research on a national level which has found more affluent people are more likely to exceed recommended drinking guidelines [5–8]. However it is not surprising our findings differ from some of the national research given that the present study is on a local level in two inner-city boroughs.

Three of the classes had a significantly higher risk of harmful or dependent drinking: Class 2 ‘economically inactive renters’, Class 3 ‘economically inactive homeowners’ and Class 6 ‘professional renters’. While additionally adjusting for CMD did not explain the increased risk of harmful and dependent drinking in Classes 3 and 6, the relative risk ratio was attenuated in Class 2 ‘economically inactive renters’ when CMD was added to the model. Previous research by our group found this class to have the highest CMD prevalence (over 40%) [21]. There is evidence that mental health drives changes in alcohol consumption [29], so one explanation is that alcohol is being used to self-medicate in this group. However it is not possible to confirm the direction of causality in this cross-sectional analysis, and a systematic review found it is more probable that alcohol use disorders precede mental illnesses such as depression [30].

While Class 2 ‘economically inactive renters’ could be considered to be the least socially advantaged class we identified, Classes 3 and 6 had markers of social advantage (high education levels and little debt, plus high home ownership in Class 3 and high incomes in Class 6) yet experienced the highest likelihood of drinking at harmful or dependent levels. These associations are not apparent from looking at the component SES variables on their own (S1 Table), where harmful and dependent drinking was common among individuals not in work, in debt, and with less secure housing. This indication that drinking at harmful or dependent levels is most prevalent in different social groups that are quite distinct from each other suggests there may be differing underlying drivers of this common health behaviour across these different groups. This is an advantage offered by the latent class analysis approach and is also not something that has been clearly observed in other studies or on a national level.

Strengths of this study include that this is the first study to our knowledge to look at the alcohol data from SELCoH in detail. We replicated some of what has been identified on a national level [5,6] in identifying some evidence of increased risks of harmful and dependent drinking in more disadvantaged groups, with this study suggesting these patterns persist at a local level and are not explained by regional differences. An important methodological strength is that we took an intersectional approach to account for the fact that multiple disadvantage is often experienced, rather than considering different aspects of disadvantage in isolation. In addition, the SES measure we specified was categorical rather than ordinal or continuous, and therefore offers a different perspective on inequality from other approaches which usually consider social gradients. This is a method that could be used more in further research into health inequalities. The improved understanding of at-risk groups offered by this segmentation approach can inform strategies for identification and health promotion.

Limitations of this study include the fact that all the classes had small numbers of participants in the highest drinking risk category (comprised of 169 harmful drinkers and 46 dependent drinkers in total). With over 1,000 participants, SELCoH II is a large survey considering the small geographical area and is broadly representative of the target population, however lack of statistical power may have limited our ability to detect differences between groups. We also used the AUDIT as a measure of higher risk drinking which is a widely-used and validated screening tool for alcohol use disorders. However more objective measures of alcohol use or harm such as biomarkers or alcohol-related hospital admissions or mortality could also have been used. We were also using the second wave of a repeated survey and there was some loss to follow-up (response rate 73%) and it is known that non-response bias does influence survey estimates of alcohol consumption (for example [31]]. However we mitigated this as far as possible by using weights account for non-response bias and to make the sample representative of the target population. Finally, we used modal class assignment for the distal outcome analysis rather than taking into account the posterior probability of class assignment (for example using the DCAT option in Mplus). This was justified in this study since the average posterior class probabilities and odds of correct classification were high, we conducted a thorough sensitivity analysis, and the approach taken also permitted the use of survey weights and adding covariates to the regression model.

By identifying distinct socio-economic groups in a large inner-city sample, we found important differences in harmful and dependent drinking in different classes, only some of which were explained by common mental disorder. In this sample we did not identify important differences in drinking at hazardous levels, but there were strong associations in between class membership and harmful and dependent drinking that were suggestive of differing underlying drivers of drinking across these different groups. This suggests that a nuanced understanding of alcohol use and problems is necessary to understand the inequalities in alcohol harms and to target public health interventions.

## Supporting information

S1 Table SES components used in the latent class analysis and AUDIT category. (DOCX)

S2 Table Sensitivity analysis to test the impact of not accounting for the posterior probability of class assignment in the regression models. (DOCX)
