# Supplementary material for: Associations between interrelated dimensions of socio-economic status, higher risk drinking and mental health in South East London: A cross-sectional study
Source: PLoS One. 2020 Feb 14;15(2):e0229093. doi: 10.1371/journal.pone.0229093 (PMC7021306; doi:10.1371/journal.pone.0229093)
Supplement: S1 Table — (DOCX) [file pone.0229093.s001.docx]

**Supplementary Table 1**

**SES components used in the latent class analysis and AUDIT category**

| **Socio-economic status component** | | ***Proportion of  overall sample*** | **Proportions in each drinking category** | | |
| --- | --- | --- | --- | --- | --- |
|  |  |  | **Low risk**  **(n=837)** | **Hazardous**  **(n=169)** | **Harmful/ dependent**  **(n=46)** |
| **Income and occupation** | **Employment status** |  |  |  |  |
|  | Employed (full or part time) | *60%* | 59% | 65% | 52% |
|  | Student | *11%* | 10% | 13% | 13% |
|  | Unemployed | *10%* | 9% | 14% | 13% |
|  | Other: sick/disabled/retired/carer | *19%* | 22% | 8% | 22% |
|  | **Occupational grade** |  |  |  |  |
|  | SOC I-II | *37%* | 34% | 48% | 33% |
|  | SOC III-M/NM | *15%* | 16% | 12% | 11% |
|  | SOC IV-V | *9%* | 9% | 6% | 9% |
|  | SOC not assigned | *40%* | 41% | 35% | 48% |
|  | **Income (gross annual household)** |  |  |  |  |
|  | £0–£12,097 | *18%* | 19% | 14% | 20% |
|  | £12,098–£31,494 | *24%* | 27% | 13% | 26% |
|  | £31,495+ | *58%* | 54% | 73% | 54% |
|  | **Benefit receipt (excl. state pension/child benefit)** |  |  |  |  |
|  | No | *76%* | 74% | 83% | 70% |
|  | Yes | *24%* | 26% | 17% | 30% |
|  | **Debt (excl. mortgage)** |  |  |  |  |
|  | No debt in past year | *84%* | 84% | 89% | 58% |
|  | Debt | *16%* | 16% | 11% | 42% |
| **Housing** | **Tenure** |  |  |  |  |
|  | Own outright/mortgage | *38%* | 38% | 40% | 21% |
|  | Private rented | *24%* | 22% | 30% | 36% |
|  | Social housing | *33%* | 35% | 25% | 33% |
|  | Rent free | *5%* | 4% | 5% | 10% |
|  | **Moves in past 2 years** |  |  |  |  |
|  | 0-1 times | *91%* | 92% | 90% | 80% |
|  | 2+ times | *9%* | 8% | 10% | 20% |
| **Education** | **Highest educational qualification** |  |  |  |  |
|  | No qualifications/GCSE (or equiv.) | *23%* | 24% | 17% | 29% |
|  | A-levels (or equiv.) | *26%* | 27% | 25% | 21% |
|  | Degree or above (or equiv.) | *50%* | 48% | 58% | 50% |

Data from 1,052 adults, weighted to account for complex survey design and non-response
