# Supplementary material for: Associations between interrelated dimensions of socio-economic status, higher risk drinking and mental health in South East London: A cross-sectional study
Source: PLoS One. 2020 Feb 14;15(2):e0229093. doi: 10.1371/journal.pone.0229093 (PMC7021306; doi:10.1371/journal.pone.0229093)
Supplement: S2 Table — (DOCX) [file pone.0229093.s002.docx]

**Supplementary Table B**

**Sensitivity analysis to test the impact of not accounting for the posterior probability of class assignment in the regression models**
